# Riboregulation in Nitrogen-Fixing Endosymbiotic Bacteria

**DOI:** 10.3390/microorganisms8030384

**Published:** 2020-03-10

**Authors:** Marta Robledo, Natalia I. García-Tomsig, José I. Jiménez-Zurdo

**Affiliations:** 1 Intergenomics Group, Departamento de Biología Molecular, Instituto de Biomedicina y Biotecnología de Cantabria, CSIC-Universidad de Cantabria-Sodercan, 39011 Santander, Spain; 2 Structure, Dynamics and Function of Rhizobacterial Genomes (Grupo de Ecología Genética de la Rizosfera), Estación Experimental del Zaidín, Consejo Superior de Investigaciones Científicas (CSIC), 18008 Granada, Spain

**Keywords:** rhizobia, *Sinorhizobium (Ensifer) meliloti*, α-proteobacteria, legumes, sRNA, non-coding RNA, dRNA-Seq, RNA-binding proteins, RNases

## Abstract

Small non-coding RNAs (sRNAs) are ubiquitous components of bacterial adaptive regulatory networks underlying stress responses and chronic intracellular infection of eukaryotic hosts. Thus, sRNA-mediated regulation of gene expression is expected to play a major role in the establishment of mutualistic root nodule endosymbiosis between nitrogen-fixing rhizobia and legume plants. However, knowledge about this level of genetic regulation in this group of plant-interacting bacteria is still rather scarce. Here, we review insights into the rhizobial non-coding transcriptome and sRNA-mediated post-transcriptional regulation of symbiotic relevant traits such as nutrient uptake, cell cycle, quorum sensing, or nodule development. We provide details about the transcriptional control and protein-assisted activity mechanisms of the functionally characterized sRNAs involved in these processes. Finally, we discuss the forthcoming research on riboregulation in legume symbionts.

## 1. Introduction

Some soil-dwelling species of the large classes of α- and β-proteobacteria, collectively referred to as rhizobia, establish mutualistic symbioses with legumes. The outcome of these interactions is the organogenesis of specialized nodule structures on the roots or, less frequently, the stems of their specific host plant. Invading rhizobia colonize nodules intracellularly and differentiate to bacteroids that achieve the nitrogenase-mediated reduction of the atmospheric dinitrogen to ammonia for the benefit of the plant. Symbiotic nitrogen (N) fixation thus renders plant growth independent of exogenously applied combined N, which is commonly provided to crops in the form of pollutant and costly chemical fertilizers. Besides this doubtless agronomical and ecological significance, the rhizobia–legume symbiosis provides a complex biological experimental model to explore the molecular mechanisms underlying bacterial adaptations during chronic intracellular infection of eukaryotic hosts [1,2,3].

The saprophytic and symbiotic competence of free-living rhizobia in soil is largely determined by their capacity to cope efficiently with the abiotic variables shaping this environment, e.g., oligotrophy, drought, salinity or acidity, which are known to influence negatively the physiology of both symbiotic partners and the N fixation process itself [4]. Competitive rhizobial strains actively colonize the rhizosphere of their compatible legume and initiate infection upon synthesis of lipo-chitooligosaccharide signal molecules (i.e., Nod factors) in response to species-specific root exuded flavonoids [3,5]. Nod factor signaling triggers major nodule developmental pathways and root hair invasion through tubular structures made of plant cell-wall material known as infection threads. Besides Nod factors, other signaling molecules of bacterial origin such as surface polysaccharides or effector proteins selectively contribute to the infection of certain legume species by their rhizobial partners [6]. During early symbiotic infection, invading rhizobia elicit a defense response in the host, which is initially featured by the release of reactive oxygen species (ROS) [7]. Compatible rhizobia counteract and survive this oxidative stress by specific mechanisms, being subsequently delivered from the branched infection threads into the cells of the nodule primordia. Inside the plant cells, rhizobia differentiate into N-fixing polyploid bacteroids that end up surrounded by a membrane of plant origin. In some legume lineages, irreversible terminal bacteroid differentiation is directed by plant-secreted nodule-specific cysteine rich (NCR) peptides [8]. Finally, mature bacteroids sense the microoxic environment inside the plant nodule, driving expression of the nitrogenase-coding gene clusters. This complex rhizobial lifestyle demands an adaptive flexibility that is supported by both large genomes and continuous gene expression shifts during host infection, cell cycle progression or in response to nutrient availability [9,10]. To date, symbiotic regulatory networks in rhizobia have been studied almost exclusively from the perspective of the transcriptional control orchestrated by proteins, i.e., transcription factors and alternative RNA polymerase holoenzymes (σ factors) [11]. Indeed, the inventory of genes encoding transcription-related proteins is particularly rich in rhizobial genomes. However, proteins are far from being the only players in the regulation of gene expression in bacteria.

**Figure 1:**
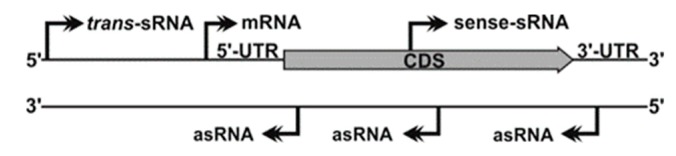
The prokaryotic non-coding transcriptome as revealed by dRNA-Seq. Identified Transcription Start Sites (TSSs; shown by arrows) can be assigned to mRNA and the different sRNA types (*trans*-sRNAs, asRNAs and mRNA-derived sense-sRNAs). See text for further details.

The development of high-throughput sequencing technologies has revolutionized our classic view of the prokaryotic transcriptome. Different, well-established strategies for the generation and deep-sequencing of cDNA libraries (RNA-Seq) allow for genome-wide mapping of transcription start sites (TSS), thus uncovering unexpected operon structures and novel transcribed regions systematically overlooked in the primary genome annotation [12] (Figure 1). Many of these newly discovered transcripts are not translated into proteins, but fulfil important regulatory roles, especially in the adaptation of bacteria to fluctuating environments [13]. Bacterial non-coding transcriptomes typically consist of heterogeneous populations of 50 to 250 nt-long RNA species (sRNAs) with diverse biogenesis pathways and regulatory activity mechanisms. Some untranslated regions at 5’-ends (5’-UTRs) of mRNAs sense shifts in temperature (RNA thermometers) or intracellular levels of certain metabolites (riboswitches) to modulate transcription and/or translation of the downstream coding sequence in a structure-dependent manner. Few other sRNAs mimic sequence or structural DNA or RNA motifs recognized by specific proteins to outcompete them at their cognate cellular targets. Nonetheless, the activity of most sRNAs thus far characterized relies on base pairing interactions to fine-tune translation and/or turnover rates of mRNA targets that can be either antisense-transcribed (asRNAs) or encoded in *trans* (*trans*-sRNAs). Complementarity between the asRNA and its mRNA target is perfect and may extend over regions even longer than 200-nt. Conversely, regulation by *trans*-sRNAs typically involves short and discontinuous series of complementary nucleotides in both molecules, requiring the assistance of proteins (e.g., Hfq) as RNA matchmakers [14]. Regardless of the type of sRNA involved in regulation and the possible effect on translation, formation of RNA duplexes through base pairing usually promote degradation of the mRNA targets by cellular ribonucleases (e.g., RNase E or RNase III). RNA-mediated post-transcriptional regulation of gene expression is ubiquitous in bacteria, influencing virtually any cellular process, e.g., stress responses, biofilm formation, quorum sensing, and other virulence traits [13].

Research on prokaryotic riboregulation has been pioneered in the early post-genomic era by work on the model bacterium *Escherichia coli*. Soon afterwards, studies in related enterobacteria (i.e., *Salmonella* spp.) and other clinically relevant microbes revealed the unprecedented prominent roles of sRNAs in the establishment of host-pathogen interactions [13]. In the last decade, functional RNomics is continuously providing new paradigms to the contribution of sRNAs to the ecological specializations of phylogenetically distant bacteria with complex lifestyles, bringing RNA to the forefront of microbial research. In legume symbionts, the knowledge about riboregulation is still scarce and rather limited to *Sinorhizobium meliloti*, the symbiotic partner of the forage legume alfalfa (*Medicago sativa* L.) and other related *Medicago* species [15,16,17]. Here, we summarize and discuss known insights into the structure, function and mechanistic principles of the rhizobial non-coding transcriptome.

## 2. Deciphering the Rhizobial Non-Coding Transcriptome: From Comparative Genomics to RNA-Seq

Non-protein coding genes largely escape classical genetics screens and the primary annotation of a single bacterial genome sequence, which is essentially limited to the prediction of open reading frames (ORFs), tRNAs, and rRNAs. Before the advent of high-throughput sequencing, computational comparative genomics was thus the tool of choice to identify conserved regions with putative functions in the unannotated portions of the genome. Accordingly, pioneering seminal genome-wide searches for sRNAs in rhizobia relied on the comparison of intergenic sequences (i.e., genomic regions between ORFs; IGRs) from phylogenetically close species to unveil sRNAs of the *trans*-acting class. Specifically, three studies published almost concurrently used the IGRs from the *S. meliloti* reference strain 1021 (Rm1021) as queries to interrogate the genomes of α–proteobacterial relatives, i.e., plant symbionts (e.g., *Mesorhizobium loti*, *Rhizobium etli* or *R. leguminosarum* bv. *viciae*), phytopathogens (*Agrobacterium tumefaciens*) and animal pathogens (*Brucella* species) [18,19,20]. All three searches combined genomic comparisons with other known features of *trans*-sRNAs, namely association with orphan transcription signatures (promoter motifs and/or Rho-independent transcriptional terminators) and conservation of thermodynamically stable secondary structures. Collectively, these approaches predicted more than a hundred IGRs in the three replicons of the Rm1021 genome (chromosome, and pSymA and pSymB megaplasmids) putatively encoding a *trans*-sRNA. Northern blot hybridization of total RNA, RACE (Rapid Amplification of cDNA Ends) mapping of transcripts boundaries and/or microarray probing experimentally confirmed that a few dozens of these candidate IGRs did express sRNA species from independent transcription units. These studies also anticipated the symbiotic and/or stress-dependent expression of subsets of the identified sRNAs. In the absence of further functional insights, these *trans*-sRNAs were initially referred to as either Smr [20], Sra [18] or Sm [19] (Table 1). A similar combination of in silico searches and experimental approaches also delivered the first inventories of sRNAs expressed by *R. etli* CFN42 (termed ReC sRNAs), *Bradyrhizobium japonicum* USDA110 (BjrC sRNAs) and *M. huakuii* 7653R (MH_s sRNAs) [21,22,23]. These rhizobia are the symbiotic partners of common bean (*Phaseolus vulgaris*), soybean (*Glycine max*), and milkvetch (*Astragalus sinicus*), respectively.

Inherent limitations to both computational searches and microarray designs necessarily biased these early genome-wide screens for sRNAs in rhizobia to the identification of putative intergenic, *trans*-acting, conserved riboregulators. Straightforward experimental identification of TSS associated to coding sequences, untranslated mRNA regions, and non-coding RNA genes in the prokaryotic genomes is now feasible with the implementation of oriented differential RNA-Seq (dRNA-Seq) [24] or Cappable-Seq [25] strategies. In particular, dRNA-Seq surveys rediscovered the early-identified sRNAs in *S. meliloti* and *B. japonicum* and further uncovered the complex rhizobial transcriptomes by adding hundreds of unknown *trans*-sRNAs, as well as thousands of mRNA-derived sRNAs and asRNAs [22,26,27,28,29]. Other RNA-Seq studies are conceived to profile specific subpopulations of cellular transcripts supposedly enriched in sRNAs, e.g., RNA species co-immunoprecipitated with the major bacterial RNA chaperone Hfq [30,31]. However, this approach resulted in a minor addition to the sRNA landscape revealed by deep-sequencing of *S. meliloti* total RNA [32].

Prokaryotic gene prediction pipelines such as EuGen-P have incorporated the novel gene structural features uncovered by dRNA-Seq for the accurate reannotation of the *S. meliloti* (strains Rm1021 and Rm2011) and *B. japonicum* USDA110 genomes [27,28,29]. Similar in silico workflows can be used to predict sRNA genes in any bacterial genome [33]. Even though dRNA-Seq mostly serves annotation purposes, comparison of transcripts levels in some datasets identified differentially expressed sRNAs in free-living and nodule endosymbiotic bacteria [26,28,29,32]. In this regard, it is noteworthy the identification of nodule-expressed sRNAs by RNA-Seq based profiling of RNA derived from each developing zone of indeterminate nodules induced on the model legume *M. truncatula* by Rm2011 [34]. On the other hand, comprehensive mapping of TSS in Rm2011 and USDA110 has facilitated the prediction of motifs putatively recognized by alternative σ factors such as RpoE2 (σ^E2^) or RpoN (σ^54^) in the promoter regions of some of the identified sRNAs, thus placing these RNA regulators in major stress response and/or symbiotic regulons [27,28,29]. The integration of the updated genome annotation files, primary expression profiles, and promoter predictions (Table 1) provides a solid resource for the forthcoming investigation of the function of sRNAs in plant symbiotic bacteria.

## 3. Conservation of Rhizobial sRNAs: α-Proteobacterial sRNA Families

Reverse comparative RNomics contributes to unravel sRNA function by identifying either functionally characterized homologs to the query transcripts or conservation patterns potentially related to the particular lifestyle of phylogenetically close bacterial species. Homology predictions typically rely on stochastic covariance models (CMs) capturing both sequence and secondary structure (folding) conservation from a multiple alignment to the query sRNA. CMs can be automatically generated by INFERNAL, which builds RNA families collected by the Rfam database (https://rfam.xfam.org/) [35]. Regardless of their assignment to specific family models, a handful of chromosomally encoded rhizobial sRNAs that occur ubiquitously and exert housekeeping functions in bacteria can be unequivocally identified by the sole comparison of the primary nucleotide sequence. This set includes:

- RNase P (Rfam family model RF00010), which is the ribozyme that cleaves off 5’-extra sequences on tRNA precursors [36].

- tmRNA or SsrA (RF00023), which has a dual function as transfer and mRNA, operating in *trans*-translation for stalled ribosome recycling [37]. Of note, tmRNA has been the only rhizobial sRNA identified experimentally by a random mutagenesis screen for symbiotic genes in *B. japonicum* [38]. The *S. meliloti* tmRNA homolog is expressed in a growth- and stress-dependent manner as an unstable precursor, further processed into two readily detectable 214 and 82 nt-long RNA species likely derived from the mRNA and tRNA domains of the primary transcript [39].

- The 4.5S RNA (RF00169), which associates with the multidomain Ffh protein, in the bacterial SRP (small signal recognition particle) ribonucleoprotein complex, universally required for cotranslational protein targeting [40].

- The 6S RNA or SsrS (RF00013) RNA, which relies on an open promoter-like structure to counteract the activity of the σ^70^ RNA polymerase holoenzyme by a target mimicry mechanism, thus contributing to the transcriptional reprogramming during transition from exponential to stationary growth [41]. Interestingly, the 6S RNA is also required for the proper multiplication of the pathogen *Legionella pneumophila* within macrophages [42], which could anticipate novel yet unexplored wider roles of this riboregulator in both virulence and endosymbiosis.

CM-based phylogenetic distribution of other 57 *S. meliloti trans*-sRNAs without associated functional evidence has been also addressed by several independent studies [43,44,45,46]. In two particular cases where high secondary structure but low sequence conservation hindered construction of the CMs, RNA families were modeled using a complementary thermodynamic matchers (TDMs) based approach [43,44]. TDMs are RNA predictors that ignore sequence conservation in some parts, rather scoring the folding energy of conserved motifs elsewhere in the structure. Altogether, these analyses collected the 57 *trans*-sRNA input sequences into 43 family models, indicating that several of these sRNA *loci* occur with different levels of paralogy in the Rm1021 genome. Distribution of the majority of these families was restricted to members of the family Rhizobiaceae within α-proteobacteria, including *Agrobacterium* species. Only a few chromosomally encoded sRNAs are conserved beyond Rhizobiaceae, occurring in representatives of Brucellaceae or even Bartonellaceae, Xanthobacteriaceae, Beijerinckaceae, or Bradyrhizobiaceae. In contrast, sRNA loci mapping to *S. meliloti* pSymB or pSymA are almost unique to *Sinorhizobium* spp. and *S. meliloti*, respectively, further supporting that these two megaplasmids mostly encode accessory functions, with pSymA being the most recently acquired replicon in genus *Sinorhizobium*. Overall, the distribution patterns of these α–proteobacterial sRNA families hint at a major contribution of vertical inheritance and frequent ancestral duplications events to the evolution of these class of riboregulators in α-rhizobia. Table 1 compiles known genomic, conservation and expression data of conserved housekeeping and putative regulatory *S. meliloti trans*-sRNAs.

## 4. Assigning Functions to Rhizobial *Trans*-sRNAs

**Figure 2:**
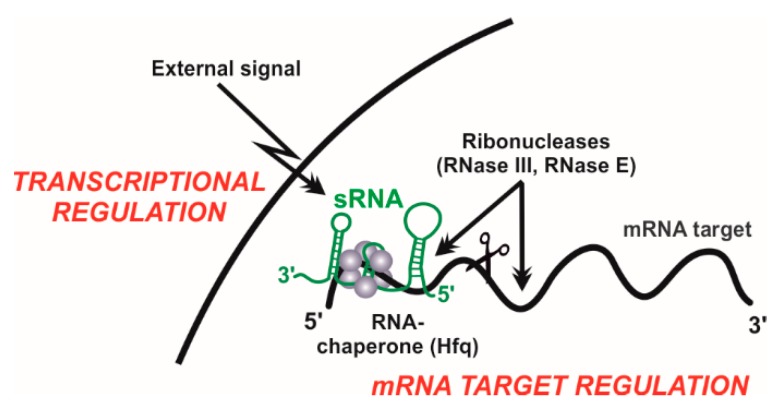
Key features of bacterial *trans*-sRNA biology. Differential regulation, target recognition and association with RNA chaperones and ribonucleases.

RNA-Seq and comparative genomics have delivered extensive repertoires of sRNAs in *S. meliloti* and related α–proteobacteria. The challenge now is to decipher the biological functions of this transcriptional output, which can be still regarded as dark matter of rhizobial genomes. In prokaryotes, a large fraction of these transcripts are catalogued as *trans*-sRNA regulators, which is an intensively investigated class of sRNAs since the early studies of the non-coding transcriptome in classical model bacteria [13]. The biology of bacterial *trans*-sRNAs is essentially characterised by their differential accumulation in response to environmental signals and target mRNA regulation by protein-assisted base-pairing mechanisms (Figure 2). Therefore, primary studies to unravel *trans*-sRNA functions must necessarily address expression profiling, transcriptional regulation, gain- and loss-of-function phenotyping, identification of mRNA targets and targeting motifs, and characterization of the associated proteins (e.g., RNA chaperones or RNases). Experimental and in silico approaches to functionally characterize *trans*-sRNAs in rhizobia are described with detail elsewhere [16,17,47,48]. Expression of *trans*-sRNAs can be tracked by classical molecular-genetics methods that include the use of promoter-reporter fusions and probing of total RNA on Northern blot membranes [47]. On the other hand, target identification typically relies on comparative genomics-based predictions of most probable conserved sRNA-mRNA base-pairing interactions (e.g., CopraRNA algorithm) followed by experimental validation of the candidates using a two-plasmid genetic reporter assay in vivo [17,47,49,50]. The latter is based on the constitutive co-expression from compatible plasmids in the same cell of the sRNA of interest and a translational fusion of the putative mRNA target to GFP, so that fluorescence of the reporter strains is related to sRNA-mediated translational repression or activation of the mRNA [17,47]. Co-expression of relevant sRNA and mRNA mutant variants in these assays enables the precise mapping of the interacting motifs in both molecules. Alternatively, *trans*-sRNA targetomes, i.e., the set of mRNA targets, can be characterized on a genome-wide scale by profiling of either transcriptome alterations upon pulse expression of the tested sRNA or the subset of mRNAs picked up by affinity chromatography using an aptamer-tagged (e.g., MS2) version of the sRNA as a bait [51,52]. Remarkably, this latter approach is also suitable to capture in vivo assembled sRNA-protein complexes, which can be further characterized by mass spectrometry analyses [48].

A combination of these strategies has been used to gain insights into the function and activity mechanisms of a handful of *trans*-sRNAs identified in *S. meliloti*. In the following paragraphs we describe how these sRNAs contribute to the regulation of cellular processes relevant to both the free-living and endosymbiotic rhizobial states.

### 4.1. Nutrient Uptake and Metabolism

Rhizobia must cope with a broad range of limiting nutrients in soil and specific nutritional needs in the rhizosphere of their host plants. Therefore, nodulation competitiveness first depends on the adequate regulation and coordination of efficient nutrient uptake and metabolism. Thus, it is not surprising the large number of ABC (ATP-binding cassette) transporters encoded by rhizobial genomes [9,53,54]. Acquisition of amino acids (aa), peptides, metal ions, or sugars through ABC transporters impacts important cellular processes such as differentiation, virulence or conjugation [55]. Remarkably, a selective suite of ABC systems is expressed in Rm1021-induced alfalfa nodules, thus supporting a specialized nutrient exchange between symbiont and host [56].

Nutrient uptake through ABC transporters primarily relies on periplasmic solute binding proteins (SBP) that determine substrate-specificity and are induced by its own ligand, typically two or more molecules [53,55]. This pervasive transcription of transporter genes suggests that rhizobia use post-transcriptional silencing mechanisms to quickly and accurately optimize metabolism with minimal energy costs. As proof of principle, the sRNA rnTrpL, which is produced from transcription attenuation of one of the three tryptophan (trp) biosynthesis operons, *trpE(G)*, directly interacts with the polycistronic *trpDC* mRNA resulting in negative post-transcriptional regulation in *S. meliloti*. Considering that trp is the most costly-to-synthesize aa and the conservation of this mechanism in *Agrobacterium* and *Bradyrhizobium* but not in *E. coli*, this precedent provides an example of how soil bacteria have evolved post-transcriptional strategies to coordinate transport and metabolism in complex ecological systems as soils [57].

Riboregulation of nutrient uptake was first described in *E. coli* and *Salmonella* by characterization of the GcvB sRNAs, which regulon potentially includes ~1% of all mRNAs in these bacteria, with prevalence of mRNAs from ABC transporters of aa and short peptides, but also containing mRNAs coding for aa biosynthesis-related proteins and transcription factors [58]. Functionally analogous riboregulators were later discovered in the α-proteobacterial species *A. tumefaciens, B. abortus* and *S. meliloti*, and were termed AbcR sRNAs (ABC transporter RNA) [59,60,61,62,63]. *S. meliloti* Rm1021 encodes three AbcR homologs that belong to the αr15 sRNA family, whose members have partial homology to the SuhB sRNA (Rfam model RF00519) [46]. AbcR1 and AbcR2 are Hfq-binding 120 and 114 nt-long transcripts, respectively, that are encoded in tandem in the IGR flanked by the protein-coding genes SMc01226-*lsrB* (Table 1). Their predicted secondary structures consist of three stem loops (SLs) with two CU-rich anti-Shine Delgarno (aSD) motifs in unpaired regions of the molecules. These motifs are well conserved in AbcR homologs and are putatively responsible for the interaction with the ribosome binding site (RBS) and flanking nucleotides in mRNA targets, which commonly interferes with translation and promotes mRNA decay. The predicted lists of targets for both *trans-*sRNAs are enriched in ABC transporter mRNAs (35–45% of the top scored). Proteomics and further genetic verification identified *prbA*, SMa0495 and *livK*, all coding for the SBP of aa transporters, as common targets of these sRNAs- except *livK* that is specifically repressed by AbcR1 [32,62].

AbcR1 and AbcR2 expression patterns are divergent, suggesting differential transcriptional regulation from independent promoters. Indeed, both 5’-end RACE mapping and dRNA-Seq consistently identified a TSS for each transcript [20,27]. AbcR1 is accumulated in exponentially growing bacteria and in the nodule invasion zone II, which accommodates undifferentiated actively dividing rhizobia. In contrast, AbcR2 is highly induced in late stationary phase and other stress conditions, but not in nodules [62]. The molecular determinants of the transcriptional regulation of these *S. meliloti* sRNAs are unknown. Analysis of the *abcR2* promoter shows high conservation of motifs recognized by the heat-shock σ factor RpoH, whereas the *Brucella abcR1* is directly and positively regulated by the LsrB homolog VltR, encoded by the neighbor gene. Interestingly, while VltR is involved in virulence, LsrB is critical for the successful symbiosis in alfalfa plants by suppression of host defense responses that contribute to nodule premature senescence [64,65,66,67]. Neither AbcR1 nor AbcR2 are required for the competitive and efficient nodulation of *S. meliloti* on alfalfa roots or free-living growth. However, AbcR homologs in *Brucella* or *Agrobacterium* are involved in virulence [61]. Notably, whereas *S. meliloti liv* ABC system has no contribution to symbiosis, its *bra* homolog in *R. leguminosarum* is needed for a Fix+ phenotype in pea [68,69,70]. Therefore, it cannot be ruled out a symbiotic relevance of AbcR sRNAs in other rhizobia–host interactions.

Proper regulation of N and carbon (C) metabolism is also required for successful bacteroid differentiation and N fixation. C storage in nodules occurs in the form of polyhydroxybutyrate (PHB) granules which are produced during the invasion process and degraded during bacteroid differentiation. PHB is likely used as a C source at this stage, being critical for symbiotic performance [71]. MmgR (makes more granules regulator) is an Hfq-dependent *S. meliloti* 77 nt-long *trans*-sRNA of the αr8 RNA family (Table 1) linked to PHB metabolism. MmgR homologs have been also identified in *S. fredii, R. leguminosarum* bv. *viciae* and *R. etli* [45]. The predicted MmgR secondary structure shows three SLs and partial homology to SuhB sRNA. An aSD motif within the first loop is predicted to be involved in targeting, but no direct mRNA target of this sRNA has been described to date [45]. MmgR accumulates preferentially upon entry of bacteria into stationary phase when grown in defined minimal medium. In addition, it is induced by nitrate-limiting conditions and repressed by addition of tryptone or aa to the medium. The transcription of *mmgR* is induced by the global N regulator NtrC, while it is repressed by AniA, a regulator of C flow. As revealed by the loss-of-function phenotype, MmgR preserves synthesis of both PHB-related phasin proteins (PhaP1 and PhaP2) under conditions of C surplus, thereby limiting intracellular accumulation of PHB granules. Together, these data suggest that *mmgR* expression depends on cellular N and C status [72,73]. However, lack of the PHB synthase coding gene *phbC* has different effect depending on rhizobial species. Therefore, MmgR may have a different impact in other rhizobia that is worth to investigate [70,74,75,76].

### 4.2. Quorum Sensing

Bacteria are able to communicate with their pairs and adjust their expression profiles to cell density thanks to the quorum sensing (QS) response. This collective behavior system has been extensively studied in Gram-negative bacteria and it is mainly based on the synthesis of autoinducers, such as acyl-homoserine lactones (AHLs) [77]. These molecules are usually recognized by their cognate LuxR receptors, triggering gene expression changes involved in important biological processes like biofilm formation and virulence. Autoinducers diffuse easily through bacterial membranes and can also stimulate LuxI-type AHLs synthases in neighboring microorganisms, orchestrating transcriptional control. In *Vibrio*, the model microorganism to study AHLs-based QS regulation, several sRNAs modulating this process have been identified. In the absence of AHLs, *V. harveyi* expresses five Hfq-dependent homologous QS Regulatory RNAs (Qrr) that regulate multiple mRNAs, including four low cell density master regulators that repress LuxR by four distinct mechanisms [78].

In *S. meliloti*, AHLs are synthesized by the LuxI-type synthase SinI, which is regulated by other components of the Sin QS system, like SinR and ExpR, under cell density-dependent and stress conditions [79]. Accumulation of *sinI* mRNA in an *hfq* knock-out mutant together with co-immunoprecipitation with Hfq anticipated QS riboregulation in *S. meliloti* [32,80]. On the other hand, the conserved endoribonuclease RNAse E is involved in *sinI* 5’-UTR degradation, thereby influencing mRNA turnover and AHLs levels [81]. A *trans*-sRNA that regulates *S. meliloti sinI* post-transcriptionally has been also unveiled. This sRNA, initially referred to as SmelC587 (Table 1), was identified by inverse bioinformatics target predictions (i.e., using the 5´-UTR of *sinI* mRNA sequence as query) as a putative *sinI* base-pair riboregulator [82]. Abundance of SmelC587 transcript does not significantly vary with cell densities but with temperature and salt stress [26] and, accordingly, it was renamed Rhizobial Cold and Salinity stress Riboregulator 1 (RcsR1). Accumulation of this sRNA was very low in the presence of 500 mM NaCl in several Rhizobiaceae, matching the growth defects observed upon RcsR1 overproduction at high salinity conditions [82]. RcsR1 is predicted to fold into three distinct domains: two highly-conserved SLs and a less conserved region that targets the *sinI* 5’-UTR. This interaction was genetically confirmed in vivo and by in vitro by RNA degradation assays using native RNase E. Interestingly, the results show that RcsR1 competes with the endonuclease for *sinI* 5’-UTR cleavage site, impairing SinI translation [82]. This work represents an example of negative sRNA-mediated modulation of key bacterial processes under stress signals. Remarkably, RcsR1 is transcribed from an identical position to the *trpL*-derived attenuator RNA rnTrpL. Ectopic RcsR1 overproduction results in *trpDC* mRNA level decrease as rnTrpL down-regulates *sinI*, suggesting a co-regulation of both QS and tryptophan biosynthesis [57].

### 4.3. Cell Cycle

**Figure 3:**
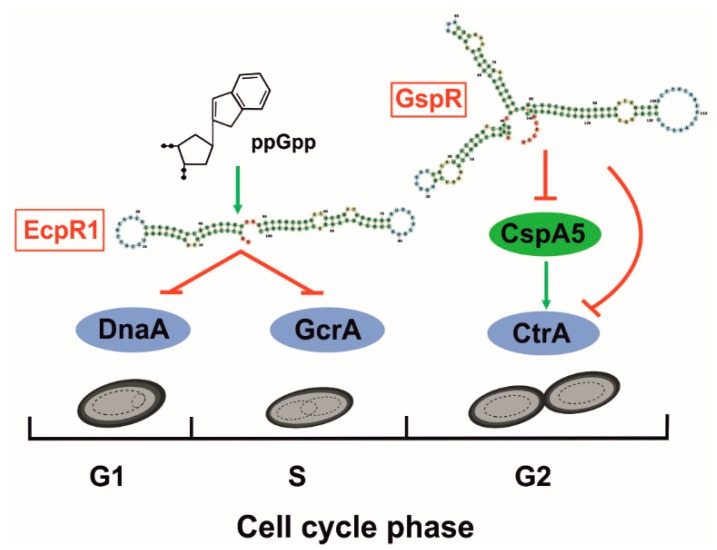
Activity mechanisms of *S. meliloti* cell-cycle *trans*-sRNAs. EcpR1 (left) is transcriptionally induced by the alarmone ppGpp and represses the early cell cycle transcriptional regulators DnaA and GcrA, restraining transition to G2 phase. GspR (right) regulates the expression of the gene encoding the essential cell division protein CtrA, preventing cytokinesis, and the cold-shock protein homolog CspA5, which is a CtrA activator.

Cell cycle progression has been widely studied in α-proteobacteria, in which there is a single chromosomal replication event per cycle (Figure 3). Mechanisms to control chromosome replication and cell division in fluctuating environments are of critical relevance for bacterial survival [83]. Accordingly, rhizobia completely rewire cell cycle during the establishment of symbiosis with leguminous plants [34,84]. Control of α-proteobacterial cell cycle relies on several conserved transcription factors regulating the main stages [10]. Among them, DnaA mediates replication initiation of *S. meliloti* chromosome and GcrA has been reported to control expression of several genes involved in the segregation machinery during S-phase in *Caulobacter* [85,86]. Interestingly, *gcrA*-depleted cells resemble bacteroid genome content and morphology in *S. meliloti* [87], suggesting a similar role of GcrA in cell cycle and also in bacteroid differentiation. Finally, the master cell cycle regulator CtrA controls genes involved in cell division and inhibits replication reinitiation [88]. This essential protein is tightly controlled and negatively regulated, together with GcrA, by the widely-studied plant peptide NCR247, which suggests its involvement in differentiation of N-fixing bacteroids [84]. To date, two *S. meliloti trans*-sRNAs have been related to the adjustment of cell cycle progression under adverse conditions by different mechanisms (Figure 3); EcpR1 represses *dnaA* and *gcrA* expression [87] whereas GspR contributes to *ctrA* silencing [89].

Target predictions for conserved sRNA families in related α-proteobacteria showed an enrichment of cell cycle-related mRNAs for EcpR1 (formerly SmelC291; Table 1), whereas reverse searches using the 5´-UTR of the *ctrA* mRNA as a bait ended up with the identification of GspR (formerly SmelC775) as possible CtrA riboregulator. EcpR1 homologs are broadly distributed among Rhizobiales, whilst GspR occurrence is likely restricted to the genus *Sinorhizobium* [43]. The name of both *trans*-sRNAs stand for the main phenotypes associated to their gain-of-function. EcpR1 overexpression results into an elongated cell phenotype, along with cell filamentation and genome endoduplication, mimicking the phenotypes of bacteroid and GcrA-depleted cells [87]. Over accumulation of GspR hampers cell growth (growth stop phenotype) in accordance with cell cycle progression defects [89]. Interestingly, these effects are also visible in related species upon overproduction of the respective sRNA homologs. Deletion mutants do not show clear growth phenotypes, however, EcpR1- and GspR- *S. meliloti* derivatives are outcompeted by their parent strains when co-cultured, suggesting that both sRNAs confer a fitness advantage to bacteria. Expression of both EcpR1 and GspR is induced in growth-limiting stationary phase in rich media and is not detected in mature nodules [87,89]. EcpR1 is preceded by a σ^70^-dependent promoter and this sRNAs is also activated by other stresses like cold and heat shocks or nutrient deprivation, stimulated by the stringent response signal ppGpp. GspR strongly accumulates in bacteria grown in minimal medium. In agreement with in silico predictions and profiling of their dependent proteomes, EcpR1- and GspR-mediated regulation of cell cycle-related genes was experimentally validated by an eGFP-based genetic reporter system [47]. However, EcpR1 and GspR use different targeting mechanisms for mRNA regulation. The two EcpR1 confirmed mRNA targets (*dnaA* and *gcrA*) base pair to the same GC-rich conserved SL in the sRNA, whereas GspR represses expression of *ctrA* and *cspA5* using different domains. Furthermore, considering the targeted mRNA region, *dnaA* and *cspA5* are canonically regulated by sRNA binding to the vicinity of the RBS and the start codon. However, *gcrA* and *ctrA* are silenced by a still unknown mechanism that involves base pairing to nucleotides located far upstream of the translation initiation region within the long 5’-UTR of the mRNAs. CspA5 homologs usually act as RNA chaperones facilitating translation, which is probably the observed effect of CspA5 on the long 5’-UTR of the *ctrA* mRNA. Therefore, GspR and CspA5 are arranged in a coherent feed-forward loop that adds a novel post-transcriptional layer to the intricate CtrA regulation [89]. These results anticipate that RNA-mediated post-transcriptional control of cell cycle genes may be a universal mechanism linking cell growth and stress in bacteria. From a biotechnological perspective, these cell-cycle related sRNAs might be exploited as drug targets in antibacterial therapies.

### 4.4. Nodule Development and Functioning

Several studies addressing gene expression in legume root nodules have identified highly expressed sRNAs in endosymbiotic rhizobia, thus anticipating riboregulation of bacterial symbiotic traits [21,29,34,90]. Further, differential accumulation of these transcripts in defined zones of indeterminate alfalfa nodules or in developmentally arrested nodules from *M. truncatula* mutants could be predictive of specific roles of *S. meliloti* sRNAs in early, intermediate and late symbiotic stages, e.g., infection, bacteroid differentiation or N fixation [34,91]. A small subset of these *S. meliloti* sRNAs is uniquely expressed in nodule tissues, thus suggesting yet unexplored RNA functions in regulation of genuine symbiotic pathways. However, in most cases expression in nodules concurs with stress-induced accumulation of the sRNA in free-living rhizobia. This is the case of some of the functionally characterized *S. meliloti trans*-sRNAs described above, e.g., AbcR1, MmgR, EcpR1 or GspR1, whose loss-of-function has none or modest impact in symbiosis [62,72,87,89].

To date, phenotyping of knock-out mutants has revealed putative symbiotic functions for only one chromosomally encoded *S. meliloti* regulatory *trans*-sRNA, identified in early genome-wide screens as Smr14C2 or SmelC397, and later renamed NfeR1 (nodule formation efficiency RNA) to reflect this fact [20,26,92]. NfeR1 belongs to the so-called αr14 family of α–proteobacterial sRNAs (Table 1), which is represented in most plant symbionts, phytopathogens and mammal pathogens within Rhizobiaceae, Brucellaceae and Phyllobacteriaceae bacterial families [44,46]. αr14 sRNAs share a predicted secondary structure consisting of three hairpins with hypervariable nucleotide content in the stems and the aSD motif unpaired in all the loops [44,46]. NfeR1 locus occurs in six copies in the *S. meliloti* Rm1021 and Rm2011 genomes, but in the conditions tested only one of those is transcribed to levels reliably detected by northern blot probing [92]. NfeR1 transcription is driven by a σ^70^-dependent promoter containing several nucleotide residues that are well-conserved in promoter regions of αr14 sRNAs. This conserved motif is the determinant of both differential expression and high transcription rates, which render NfeR1 as one of the transcripts with highest coverage scores among the identified *S. meliloti* sRNAs by RNA-Seq surveys [26,27,29,34]. Intracellular NfeR1 levels are particularly high when bacteria are grown in defined glutamate/mannitol medium, upon salt shock and in all steps of the symbiotic interaction between *S. meliloti* and alfalfa, i.e., rhizoplane colonization, root hair infection, bacteroid differentiation and symbiotic N fixation [34,92]. Linked to this expression profile, NfeR1 loss-of-function compromises *S. meliloti* survival in hypersaline medium, nodule development and overall symbiotic efficiency [92]. Nodules induced by a *S. meliloti nfeR1* deletion mutant on alfalfa roots are apparently colonized by fully differentiated bacteroids, but look round shaped rather than elongated, and smaller than wild-type indeterminate mature nodules. Their histology resembles that of nodules developed by *M. truncatula* mutants in marker genes for intermediate and late symbiotic stages [91,93] or induced by *S. meliloti* derivatives engineered to overexpress N assimilation pathways concurrently with symbiotic N fixation [94]. In silico searches for NfeR1 mRNA targets predict that most of the regulatory potential of this sRNA resides in the three conserved aSD motifs, which are indistinctly suited for base-pairing with the translation initiation region of multiple mRNAs, most coding for transport proteins. NfeR1-mediated post-transcriptional silencing of some of these targets and the redundant function of the three identical regulatory motifs have been genetically confirmed [92]. Together, these findings anticipate a pivotal role of NfeR1 in reprogramming important rhizobial metabolic pathways during the free-living to symbiotic transition.

Nodule development is largely controlled by complex organogenesis programs induced by rhizobia in their compatible legume host [95]. In this regard, a very recent study has shown that 18-24 nt-long sRNAs primarily derived from tRNA 3’-ends (tRFs) accumulate in nodules, acting as unprecedented rhizobial signals that modulate soybean nodulation by *B. japonicum* [96]. These miRNA-like tRFs interfere with the ARGONAUTE1-dependent RNAi machinery to silence plant host target genes involved in root hair curling and nodule formation. This breakthrough report unveils a novel biogenesis pathway for rhizobial sRNAs and provides an example of cross-kingdom communication through RNA trafficking that undoubtedly merits further investigation in this and other symbiotic associations.

## 5. Antisense Transcription in *S. meliloti*

Several studies on *S. meliloti* anticipate a broad impact of *cis*-acting asRNA regulators on both the free living and host-associated lifestyles of this bacterium [26,27,97]. asRNAs are transcribed opposite to mRNA loci and may overlap either the 5´/3´-UTRs or the coding region of the message (Figure 1). The long and perfect complementarity between asRNAs and their targets favors duplex formation, which can influence transcription, translation and/or turnover rates of the mRNAs. Deep-sequencing of prokaryotic transcriptomes is continuously uncovering pervasive antisense transcription, which likely contributes to the posttranscriptional buffering of gene expression levels [98]. Such RNA-Seq surveys have revealed that *S. meliloti* expresses more than 3000 asRNAs that are linked to ~35% of the predicted protein-coding genes [26,27]. Nonetheless, functional asRNAs in *S. meliloti*, like in other microorganisms, were firstly reported associated to extrachromosomal replicons [99].

A systematic screening strategy to identify novel RNA regulators of N fixation revealed that both asRNAs and *trans*-sRNAs are overrepresented (41% and 24%, respectively) in *S. meliloti* pSymA symbiotic megaplasmid, with asRNAs particularly biased to *nod* and *nif* genes. Further characterization of seven pSymA-borne asRNAs showed that most accumulate in response to different biological conditions, anticipating functional roles [97]. Among them, SMa_asRNA_244, antisense to the of *nodD2* 3´-UTR, is slightly upregulated at high optical densities and upon luteolin addition (30%). It is worth noting that NodD2 is the only transcriptional regulator of *S. meliloti nod* genes that is not activated by this plant flavonoid. Several asRNAs opposite to genes encoding the nitrogenase components NifE and NifK show also distinct expression profiles. SmelA031 and SMa_asRNA_277, also accumulate in early and late stationary phase of growth, respectively, while SMa_asRNA_279 responds to other stresses. Interestingly, the three transcripts are repressed in nodules and under low O_2_ and N_2_ concentrations, and plants inoculated with *S. meliloti* overexpressing SmelA031 are moderately affected in N fixation when compared to controls. Nitrogenase activity is extremely sensitive to O_2_ and needs N_2_ as substrate to function inside the nodules. Therefore, it is feasible that such post-transcriptional regulation modulates translation of this enzyme to avoid energy loss. These results first evidenced symbiotic-related asRNA regulation in rhizobia, but hundreds of rhizobial asRNAs await functional characterization. There are some works arguing that the majority of cellular asRNAs just arise from noisy transcription and are non-functional [100,101]. However, there is an increasing number of roles assigned to the widespread asRNAs, including both highly specific and global regulation such as DNA-repair and transcription interference [98]. Nevertheless, it is also advisable that, when searching for asRNAs with potential specific regulatory roles, requirements like upstream predicted promoters, high read coverage in reliable RNA-Seq profiling, and informative validated expression patterns are taken into account.

## 6. Proteins Assisting sRNA Activity

Proteins involved in RNA activity and metabolism are universal players in riboregulation at different levels [102,103,104]. For example, RNA-binding proteins of the CsrA (C storage regulator)/RsmA (regulator of secondary metabolism) family prevent translation by targeting the SD sequence of some mRNAs, and this activity is counteracted by sRNAs of the Csr/Rms class by a target mimicry mechanism [105]. Intriguingly, rhizobial genomes do not encode recognizable homologs of CsrA-like proteins. Other RNA protein partners act as chaperones that promote unwinding of complex secondary structures, e.g., cold-shock proteins (CSPs) [106] or base-pairing between regulatory sRNAs and their targets, e.g., the well-characterized Hfq [107] and the recently discovered ProQ [108]. On the other hand, cellular ribonucleases with diverse substrate preference are the effectors of sRNA-mediated post-transcriptional gene silencing [109]. The role of RNA-binding proteins and ribonucleases in RNA-mediated regulation is best understood in the model *E. coli*, phylogenetically close enterobacteria and other pathogens [102]. Nonetheless, RNA research has also contributed to gain specific insights into the biology of these proteins in rhizobia [103,110].

### 6.1. The Rhizobial RNA Chaperone Hfq

The host factor for the replication of the Qβ bacteriophage or Hfq is encoded by 55% of the bacterial genomes sequenced so far [111]. The function of this protein as RNA chaperone extends far beyond its primary role in intracellular RNA phage multiplication, to assist regulation by sRNAs (preferentially *trans*-sRNAs) and stabilize diverse RNA species including mRNAs and tRNAs [14]. These RNA-binding features place Hfq at a node of bacterial post-transcriptional regulatory networks. All α–rhizobial genomes host an Hfq coding gene that appears to be strongly expressed and autoregulated at the translational level [112,113,114]. The rhizobial Hfq polypeptide has a C-terminal region shorter than that of its enterobacterial counterparts, but retains the core RNA-binding domains of canonical Hfqs [14,115]. This predicts similar quaternary structural arrangements of rhizobial and enterobacterial Hfq proteins into ring-shaped homohexamers that expose two distinct surfaces for RNA binding. Accordingly, *E. coli* and *S. meliloti* Hfq homologs have been shown to be functionally interchangeable [112].

Like in enterobacteria, *hfq* knock-out causes severe pleiotropic phenotypes in *S. meliloti* that include altered growth, increased sensitivity to abiotic stress, changes in the profile of QS signals, reduced competitiveness for nodulation, compromised survival of bacteroids inside nodule cells and defects in N fixation [80,114,115,116,117]. Late symbiotic deficiencies of these mutants are most likely related to the positive contribution of Hfq to the post-transcriptional control of genes encoding the master regulators of N fixation NifA and FixK in diverse free-living and symbiotic α–proteobacterial diazotrophs by mechanisms that are still poorly understood [113,114,115,117,118]. Nonetheless, this phenotypic pleiotropy can be further explained by the profound Hfq-dependent changes observed in the rhizobial transcriptome and proteome upon free-living growth, as recurrently revealed by several independent studies on *S. meliloti* [80,115,116,119]. One remarkable observation common to all these reports is the pervasive misregulation of genes encoding ABC transport proteins in bacteria lacking Hfq, with overall upregulation of those presumably involved in uptake of aa and other N sources, such as the broad specificity Aap and Bra systems [70,120]. Although probably involving both common and host-specific regulatory mechanisms, the central role of Hfq in the chronic infection of eukaryotic organisms has been also reported in plant and mammal pathogens within the α–subgroup of proteobacteria such as *A. tumefaciens* or *Brucella* species [61,121,122].

Regulation of at least part of the Hfq-dependent mRNAs is expected to involve its cognate partner sRNAs. In *S. meliloti*, genome-wide profiling of RNA species co-immunoprecipitated with a functional FLAG epitope-tagged Hfq variant (CoIP-RNA) uncovered massive binding of the protein to mRNAs [32,123]. Specifically, nearly 20% of predicted *S. meliloti* mRNAs were enriched in Hfq CoIP-RNA, most of which functionally related to major symbiotic and stress-response pathways. Many of these mRNAs, such as those encoding NifA, FixK or stress-related alternative RNA polymerase σ factors of extracytoplasmic function (e.g., RpoE2) are positively regulated by Hfq by a mechanism that probably involves protection of the transcripts from degradation by cellular ribonucleases in a *trans*-acting sRNA-independent manner [103]. Conversely, mRNAs encoding aa transporters that are predicted or experimentally confirmed targets of the functionally characterized *trans*-sRNAs AbcR1 and AbcR2 are particularly abundant among the Hfq-binding transcripts. In these cases, Hfq most likely acts promoting both *trans*-sRNA stability and base-pairing to mRNA targets to boost post-transcriptional silencing [103]. Nonetheless, only 14% of *trans*-sRNAs and 2% of asRNAs currently annotated in the *S. meliloti* genome were scored as Hfq ligands [26,27,29,32]. These findings indicate a rather more limited impact on riboregulation of the rhizobial than enterobacterial Hfq homolog, anticipating that in the legume symbionts other alternative RNA chaperones may also assist sRNA activity. One possible candidate is the recently identified *S. enterica* ProQ protein [108,124]. However, the phylogenetic distribution of this protein is more restricted than that of Hfq, being unique to *R. leguminosarum* bv. *viciae* among α–rhizobia [125]. Thus, the search for novel RNA chaperones in this group of bacteria remains open.

### 6.2. Rhizobial Ribonucleases

As major determinants of the steady-state level of all cellular transcripts, ribonucleases (RNases) are critical elements in post-transcriptional regulatory networks [126]. Decay of mRNA upon antisense interaction with regulatory RNAs commonly involves the prevalent prokaryotic endoribonucleases RNase E and/or RNase III, which are specific to single- and double-stranded RNAs (ssRNA and dsRNA), respectively [127]. In some cases, RNase E is recruited by Hfq to the interplay between a *trans*-sRNA and its target(s) as a component of a multiprotein complex called RNA degradosome that also contains a 3′-exoribonuclease (polynucleotide phosphorylase or PNPase), a RNA helicase (RhlB) and an enolase as the core proteins [126]. On the other hand, RNase III preferentially recognizes RNA duplexes, thus being a major player of gene silencing promoted by asRNAs [128]. RNA decay initiated by RNase E or RNase III results into intermediate products that are further degraded by other cellular endo- and exoribonucleases, e.g., PNPase, RNase II or RNase R [127].

Rhizobial genomes encode a set of more than 20 ribonucleases that include all the aforementioned proteins, but for the vast majority there is a lack of functional information [129]. Nonetheless, several reports have anticipated the involvement of RNase E in *trans*-sRNA stability and regulation in both Hfq-dependent and independent manner [81,82,87,130,131]. RNase III and RNase J, which is not present in *E. coli*, are both involved in rRNA maturation but their putative role in riboregulation has not been investigated yet [132,133]. RNase III encoded by the *S. meliloti rnc* gene has been biochemically and genetically characterized [134]. Its catalytic features resemble those of the *E. coli* ortholog but with different requirements for optimal activity regarding metal co-factor preference or pH tolerance that could be related to the rhizobial lifestyle. Indeed, although *rnc* deletion does not compromise viability and morphology of *S. meliloti* cells, it is detrimental for growth and overall symbiotic performance on alfalfa roots [134].

The gene annotated as *SMc01113* in the chromosome of the *S. meliloti* reference strain Rm1021 is almost ubiquitous in bacteria and has been included in the proposed minimal prokaryotic genome [135]. It encodes a putative metal-dependent hydrolase, later called YbeY, that shares predicted structural similarities with the MID domain of the Argonaute (AGO) proteins involved in eukaryotic RNA silencing, but its function has remained elusive until recently [136]. *S. meliloti* Hfq and YbeY mutants show strikingly similar free-living and symbiotic phenotypes as well as misregulation of several predicted sRNA-mRNA regulatory pairs [136,137]. These structural and phenotypic features initially hinted at an Hfq-like role of YbeY in riboregulation [136]. However, the *E. coli* YbeY ortholog was later shown to be a metal-dependent endoribonuclease that specifically acts on ssRNA [138]. Biochemical characterization of *S. meliloti* YbeY also supports a universal role of this protein as RNase [139,140]. However, unlike its enterobacterial ortholog, *S. meliloti* YbeY is also competent for cleaving dsRNA [139]. Transcriptome alterations in the *S. meliloti* YbeY mutant predict a role of this enzyme in *trans*-sRNA and asRNA mediated silencing of genes involved in nutrient uptake and symbiotic N fixation, respectively [139]. In this regard, both in vivo and in vitro evidences suggest that YbeY is required for AbcR2-dependent down-regulation of the ABC amino acid transporter *prbA* mRNA. Further supporting a role in dsRNA degradation, *S. meliloti* YbeY has been also shown to co-purify with several asRNAs and their mRNA partners, including the symbiotically-relevant *nifA* [139]. It would be interesting to test whether the alteration of asRNA-mediated regulation constitutes one of the main drivers of the symbiotic phenotype associated to YbeY activity.

## 7. Conclusions and Perspectives

**Figure 4:**
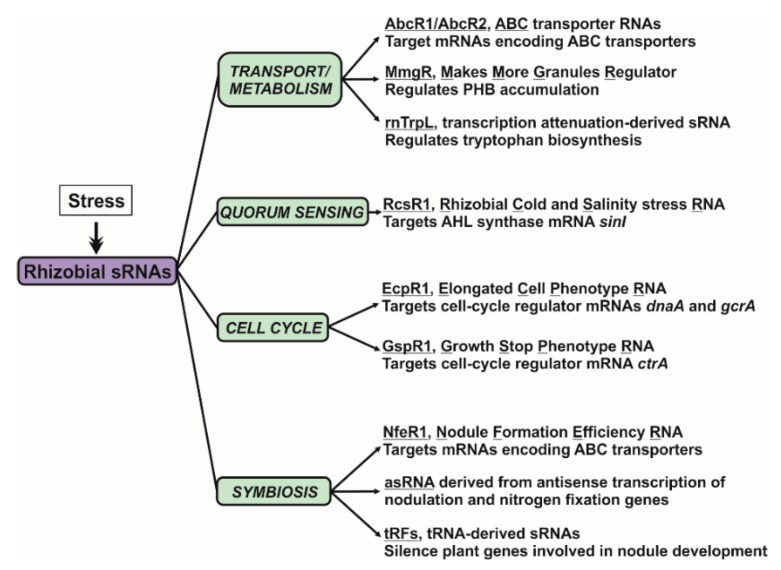
Cell processes that are riboregulated in rhizobia and the sRNAs involved.

RNA-mediated regulation of gene expression, which is ubiquitous in all living organisms, contributes to fine-tune most, if not all, prokaryotic physiological processes. However, genome annotations of the majority of bacterial species do not include sRNA genes yet, so that the structure and function of the non-coding transcriptomes remains unaccountably unexplored. Knowledge gaining on how the transcriptomes shape the functions of bacteria populating diverse niches or even establish complex relationships with other organisms is now timely and technically feasible. In plant-nodulating rhizobia, most of the knowledge about riboregulation derives from work on the alfalfa symbiont *S. meliloti*. This bacterium expresses hundreds of *trans*-sRNAs, but only a handful of those have been characterized and functionally related to free-living and symbiotic rhizobial traits such as metabolism, cell-cycle, quorum sensing or nodule development (Figure 4). Like in other bacterial species, pervasive antisense transcription also occurs in *S. meliloti*, with a remarkable bias towards symbiotically relevant genes. However, the functional significance of asRNA-mediated regulation has not been investigated further neither at genome-wide scale nor for particular symbiotic genes. Other key players in riboregulation, like RNA-binding proteins and ribonucleases remain also underexplored. Unlike in enterobacteria, the well-characterized RNA-chaperone Hfq seems to have a rather limited role in assisting *trans*-sRNA activity in rhizobia and the catalytic features of the set of predicted ribonucleases and its impact in RNA silencing are essentially unknown. Therefore, much work remains to be done to characterize the content and function of the sRNAs-associated proteomes.

The knowledge gained in *S. meliloti* should be necessarily extended to other rhizobia and symbiotic systems. Genomes of other model N-fixing symbionts must be accurately annotated using the most recent RNA-Seq protocols to uncover the primary transcriptome. The resulting catalogs of sRNA genes must be extensively compared and characterized to assess the contribution of riboregulation to the symbiotic diversity. Deciphering the biogenesis, function, and mechanistic principles of all types of sRNAs, i.e., *trans*-sRNAs, asRNAs, riboswitches, 5’/3’-UTRs, other mRNA leaders, and tRFs will contribute to explore the plasticity of RNA molecules as regulators of gene expression. Of particular interest would be to investigate the RNA trafficking between rhizobia and the plant host cells as a novel layer of dialogue between the symbiotic partners. Finally, the biotechnological implications of post-transcriptional RNA-mediated regulation for the engineering of symbiosis and other environmentally relevant systems should be also explored.

## Figures and Tables

**Table 1:** Identification, conservation and functional data of *S. meliloti* sRNAs.

								Symbionts							Pathogens		
	Name/s in *S. meliloti* 1021 and (*S. meliloti* 2011) ^a^	Genome Copies	Coordinates in *S. meliloti* 1021 Genome	Strand	Predicted Promoter Motifs [27]	Hfq Co-IP RNA [32]	Expression in Nodules ^b^ [34]	*S. meliloti*	*S. fredii*	*S. medicae*	*R. leguminosarum*	*R. etli*	*M. loti*	*B. japonicum*	*Agrobacterium sp.*	*Brucella sp.*	Others
Housekeeping	**SMc04478/tmRNA/*ssrA***	1	2291143–2291463	+	RpoD												
	**SmelC035/sra05/4.5S/SRP–RNA/*ffs* (SMc06075)**	1	259927–260032	+	RpoD RpoN		ZII/IZ/ZIII										
	**SmelC667/sra56/Smr22C/6S RNA/*ssrs* (SMc06867)**	1	2972090–2972252	-	RpoD		IZ/ZIII										
	**SmelC809/sra50/SmrC19/RNase P/*rnpB* (SMc06867)**	1	2356793–2357134	-	RpoD		IZ/ZIII										
*trans-*sRNAs	**SmelC411/sm3/sra41/smrC15/AbcR2 (SMc06510)**	3	1698731–1698617	-	RpoD RpoH1/2		IZ/ZIII										
	**SmelC412/sm3’/sra41/smrC16/AbcR1 (SMc06511)**	3	1698937–1698817	-	RpoD		ZII/IZ/ZIII										
	**SmelC397/smrC14/NfeR1 (SMc06496)**	6	1667613–1667492	-	RpoD		IZ/ZIII										
	**SmelC689/MmgR (SMc06891)**	3	3046712–3046789	+	RpoD RpoN		IZ/ZIII										
	**SmelC706/smrC45/SpeF (SMc06909)**	1	3105298–3105445	-	RpoH1/2		ZIII										
	SmelA075/smA8 (SMa6506)	5	1220693–1220808	+	RpoD		ZII/IZ										
	SmelC023/sra03/smrC7 (SMc06056)	1	201679–201825	+	RpoD RpoH1/2		ZII/ZIII										
	SmelC289/sra32/smrC9 (SMc06412)	1	1398278–1398426	-	RpoD		ZII										
	**SmelC291/sra33/smrC10/EcpR1 (SMc06416+SMc06418)**	1	1411678–1411848	+			ZII/IZ										
	SmelC671/sm84 (SMc06875)	1	2986421–2986520	+			ZII										
	SmelA099/smrA6 (SMa6570)	1	1328175–1328334	-			ZII/IZ/ZIII										
	SmelB053/smrB35 (SMb23147)	3	577730–577873	+	RpoD RpoH1/2		ZII										
	SmelC151 (SMc06261)	1	843451–843524	+			IZ/ZIII										
	**SmelC587/sm104/RcsR1 (rnTrpL?)**	1	2575832–2575947	-													
	SmelC165	1	910182–910297	+	RpoN												
	SmelC416/sm138 (SMc06519+)	1	1718814–1718919	-													
	SmelC500 (SMc06663)	1	2180099–2180274	-			ZII										
	SmelC507 (SMc06677)	1	2206348–2206579	+	RpoD RpoH1/2		ZII										
	SmelC549/sm4 (SMc06721)	1	2371597–2371855	+	RpoD RpoH1/2		ZII/IZ(ZIII										
	SmelC601 (SMc06778)	1	2625313–2625439	-	RpoE2		ZII/IZ/ZIII										
	**SmelC775/GspR1 (SMc07200)**	1	3509658–3509803	+			ZII										
	SmelA003	4	15179–15268	+													
	SmelA033/smA3a/smrA2	1	512140–512221	-	RpoD												
	SmelB003 (SMb23003)	1	30240–30345	+	RpoH1		IZ/ZIII										
	SmelB008	1	65071–65147	+	RpoE2												
	SmelB009	1	65186–65266	-	RpoH1/2												
	SmelB033 (SMb23075)	1	379319–379391	-	RpoN		IZ										
	SmelB044/smrB3b (SMb23116)	2	541771–541909	-			ZII/IZ										
	SmelB075 (SMb23231)	1	800730–800793	-	RpoH1/2		ZII										
	SmelB126/smB9 (SMb23362)	4	1325476–1325586	+	RpoE2		ZII/ZIII										
	SmelC434/sm118 (SMc06565)	1	1821211–1821366	+			ZII										
	SmelA001 (SMa6000)	1	1002–1054	+	RpoE2		IZ/ZIII										
	SmelA014 (SMa6078)	1	206669–206772	+	RpoH1/2		ZII										
	SmelA018 (SMa6091)	1	234850–234930	-	RpoE2		ZIII										
	SmelA019	1	235259–235355	+	RpoH1/2		ZII/IZ/ZIII										
	SmelA020 (SMa6094)	1	235393–235497	-	RpoH1/2		ZII/IZ/ZIII										
	SmelA054 (SMa6367)	1	911299–911374	+			ZII										
	SmelA056 (SMa6389)	1	954480–954620	-			ZII										
	SmelC032	1	241172–241247	+													
	SmelC749 (SMc07132)	1	3383055–3383121	-			ZIII										

^a^ sRNAs identified in the chromosome (SmelC) and megaplasmids pSymA (SmelA) and pSymB (SmelB). In bold, sRNAs with assigned functions as described in the text. ^b^ Nodule zones: ZII, invasion zone; IZ, bacteroid differentiation zone; ZIII, N fixation zone. Shadowed boxes stand for the presence/conservation of a given sRNA, +, positive/-, negative DNA strand.
